# Economic evaluation of an active search system to monitor the outcomes of health-related claims

**DOI:** 10.31744/einstein_journal/2020GS5129

**Published:** 2019-12-20

**Authors:** Nayara Luiza Pereira Rodrigues, Victor Zaia, Joseval Martins Viana, Paulo Roberto do Nascimento, Erik Montagna

**Affiliations:** 1 Faculdade de Medicina do ABC, Centro Universitário Saúde ABC, Santo André, SP, Brazil.; 2 Superintendência de Controle de Endemias, Secretaria Estadual de Saúde, São Paulo, SP, Brazil.

**Keywords:** Health's judicialization, Equity in access to health services, Outcome assessment (Health care), Equity in the resource allocation

## Abstract

**Objective:**

Economic evaluation of a scientific advisory program with the Public Defenders Office to mitigate the impacts of the judicialization on health in the municipality, as well as the implementation of an active follow-up program to monitor health outcomes arising from court demands.

**Methods:**

A two-step study, the first documental, retrospective, with data collection of lawsuits in the region of Barbalha (CE), Brazil, from 2013 to 2018, and the second stage, prospective and intervention, through mediation between the citizen and the Public Defenders Office, aiming to reduce the occurrence of the judicialization, and the monitoring of the health outcomes of the processes. The study adopted the Consolidated Health Economic Evaluation Reporting Standards protocol for economic health assessments. The data obtained from the processes were grouped and treated for characterization of the scenario. A comparison of the profile of the lawsuits in the period of 12 months before and after the installation of the program to delimit a complete fiscal cycle was carried out.

**Results:**

The advisory service promoted a decrease of 40% (p=0.01) in lawsuits. There was a 31% reduction in court costs (p=0.003), with medicines accounting for 33% of this amount. There was a decrease in inputs outside the *Sistema* Único *de Saúde* lists (27%; p=0.003), however there was no statistical difference among several demanding groups, suggesting an equanimous approach.

**Conclusion:**

Data from the initial survey were comparable to those reported in Brazil regarding the profile of judicial demands. In view of the scenario, the proposal proved feasible as a means to mitigate the costs of the judicialization through mediation. Finally, the initiative can serve as a model for adoption by municipalities that have characteristics similar to those presented in this study.

## INTRODUCTION

Judicialization of health care is a typical feature of the Unified Health System (SUS - *Sistema Único de Saúde*) - Brazilian National Health System, where court decisions interfere with the compliance with healthcare policies and are enforced in an unorganized way exceeding the estimated budget.^(^^1^^)^ It involves ethical issues, such as the role of the Government in the mediation of individual and collective interests and rights,^(^^2^^)^ due to the imbalance created in health planning,^(^^3^^)^ negatively affecting the system beneficiaries.^(^^4^^)^

Despite the substantial amount of academic data covering medication requests,^(^^5^^)^ characteristics of proceedings,^(^^2^^)^ mapping of lawsuits in several scenarios,^(^^6^^)^ among others, the means to obtain reliable data are decentralized, with variable access to information and lack of standardization,^(^^7^^)^ regardless of the efforts made by research groups in specific segments with expressive results.^(^^8^^)^

The main item requested in court is medication.^(^^9^^)^ On the other hand, health outcomes are rarely recorded not to mention the recognized need for further academic-scientific background to expand the support to legal decisions.^(^^10^^)^

Such decisions have been studied, and high judicial interference is acknowledged^(^^11^^)^ particularly due to technical and scientific matters ignored in the decision-making process. They refer to medical prescriptions that are noncompliant with the good prescription practices,^(^^12^^)^ despite the existing legal directions for this purpose; plaintiffs' motivations for the prescription for medicine by brand,^(^^13^^)^ questions regarding efficacy of their clinical indications^(^^14^^)^ and lack of scientific evidence in the prescriptions.^(^^11^^)^ Despite all that, medical prescription is the supreme element to support court decisions.^(^^11^^)^

Conflicts of interests are also discussed, considering the substantial volume of lawsuits filed by the private sector,^(^^15^^)^ concentrated in a limited number of physicians and lawyers.^(^^6^^)^ In spite of government efforts to mitigate the costs of fraud, corruption and embezzlements, substantial sums are misused.^(^^16^^)^ However, these types of cases must be heard by criminal courts.

This complex scenario led the National Council of Justice (CNJ - *Conselho Nacional de Justiça*) to encourage Federal and State Courts to seek technical support to ground their health-related decisions, and the development of resolutions to monitor and solve legal actions involving health care issues.^(^^17^^,^^18^^)^

Therefore, the government has means to prevent litigation from being the first option of a SUS user, including without limitation, intervention to prevent the filing of a legal action via scientific advice, conciliations before the filing of the lawsuits preventing the judicialization and the monitoring of outcomes in search of effectiveness throughout the proceeding.^(^^19^^)^

In spite of the foregoing, information technology resources are not available in most Brazilian municipalities. Currently, no technology is offered to monitor the allocation of resources, their uses and outcomes. As a consequence, despite the control and traceability of these proceedings, the investments made are not monitored. The atomization of resources reaches individuals by the capillarity of the system and poses a management challenge when creating the need for a specific and tailor-made monitoring of the system.

## OBJECTIVE

To conduct an economic assessment of the Office for Assistance in Judicialization of Healthcare, established by the Municipal Health Office in the Public Defenders Office, aiming to mitigate the impacts of judicialization of health care in a Brazilian municipality. Based on said data, the Office for Assistance in Judicialization of Healthcare is proposed as a service model for municipalities with similar sociodemographic characteristics.

## METHODS

### Experimental design

A documentary, retrospective, exploratory, descriptive, quantitative study conducted based on the collection of data from the lawsuits approved for access in the health system of the judicial district of Barbalha (State of Ceará - CE), Brazil. The Consolidated Health Economic Evaluation Reporting Standards (CHEERS) protocol was adopted for standardizing the presentation of outcomes of economic assessment in health.^(^^20^^)^

### Procedures

Description of the implementation of the Office for Assistance in Judicialization of Healthcare (NAJS - *Núcleo de Atendimento de Judicialização da Saúde*) and related assignments, comparing expenses with judicialization before (Scenario A) and after (Scenario B) its implementation. A mapping of the lawsuits was conducted, with related repercussions for service and survey of profiles of plaintiffs and requests. The costs of lawsuits filed were also estimated. Comparisons covered a 12-month period before and after its implementation to circumscribe a period of time that allowed performance of quantitative comparisons. The study was intended to cover a complete cycle of seasonal variations and a period comparable to the fiscal year. Lastly, management strategies were detailed to prevent judicialization. The study sample comprised all proceedings filed against the Department of Health in the period from January 2013 up to April 2018. Data were collected manually.

### Setting

The municipality is located in the Metropolitan Region of Cariri, which is part of the conurbation of the municipalities of Crato and Juazeiro do Norte.

The NAJS was created in April 2017, and the team comprised a nurse, a pharmacist and a social worker. Two of their assignments implemented in the first phase of the operation were the objects of this study:

– To monitor the outcomes of the health lawsuits filed in the municipality; check compliance with the judicial perspective; detect and prevent frauds and waste, and obtain data related to the need or not of maintenance of the benefit.
– To mediate the relation between the user plaintiff and the court system, proposing solutions in SUS administrative level to prevent judicialization.

Claims are examined in the presence of three NAJS professionals, the Public Defender, the plaintiff and their legal representatives. Personal and sociodemographic data, reason and type of claim, test results and past interventions, medical recommendation for the request, date of the last visit of the Health Community Agent and the last evaluation held by a specialist, all supported by evidence, are registered in a list. Within a 7-day period, the user receives the answer about the procedure to be followed in order to prevent judicialization.

### Analyzed proceedings

Analyzed proceedings covered all health-related claims filed in the municipality between January 2013 up to April 2018, which proceedings were not held in camera. The identification and classification of the cases were implemented by the Ceará State Judiciary Automation System (*e-Jud)*, by consultations made by authorized civil servants.^(^^21^^)^ The data on the monitoring of outcomes are related to legal actions filed and, as such, they are publicly available. However the identity of plaintiffs, lawyers, physicians and anyone involved in said claims was omitted.

### Cost estimates

Cost estimates were based in previously published works.^(^^1^^)^ The average amount of each pharmacological unit, supply and procedure was estimated by market research and by worksheet of maximum prices to the government. To determine the unit value of medicines, a sample of each pharmaceutical industry was used with the largest number available of pharmaceutical presentations. An average unit value was sought for each pharmaceutical unit for each treatment for the period of study, as well as for supplies and procedures.

### Active search

After evaluation and collection of data from all claims, the NAJS sought for outcomes of all, except for those held in camera. Cases that could be solved by the crossing of public official data were conducted in the office (*e.g*., death of plaintiffs). The remaining cases were visited by the NAJS. All noncompliant cases were reported was referred to the Public Defender's Office for legal action.

Unified Health System users who went to court were visited. Addresses, data related to the item requested and maintenance or modification of the plaintiff's health condition were checked. In case of modification in health condition that changed the status of what had been granted in court, the plaintiff was requested to appear at the Department of Health. After all proceedings had been analyzed, the NAJS began to monitor only cases in progress and which were compliant with both medical and legal requirements.

### Data analysis

For statistical analysis, the software Statistical Package for the Social Sciences (SPSS; IBM, 2010), version 20.0 for Macintosh was used. Data were grouped according to the variables available in the claims. Descriptive analyses were conducted based on the frequency calculation with summarized results. Descriptive statistics (mean and standard deviation), correlational (Spearman's rho) and Mann-Whitney U tests were used when comparing two groups, and the Kruskall-Wallis test for three or more groups. For the type of appropriate regression analysis of data, the normality distribution of variables was checked by the Kolmogorov-Smirnov test.

### Ethical aspects

The work was conducted according to the National Council of Health's regulations. As a documentary study involving legal proceedings, a request was made to waive application of the Informed Consent Form (ICF). The study was approved by the Research Ethics Committee, opinion no. 2.924.500, CAAE: 98718918.8.0000.0082, and authorized by the Municipal Health Department and by the Public Defender's Office of Barbalha (CE, Brazil).

## RESULTS

### Characterization of the setting

The city of Barbalha ranked seventh in the state in the general Human Development Index (HDI) and ninth in HDI-Education and fourth in HDI-Longevity.^(^^22^^)^ Regional sociodemographic characteristics are presented in table 1.

**Table 1:** Sociodemographic characterization of Cariri microregion

Municipality	Area (km^2^)	Inhabitants	DD (Inhabitants /km)	HDI	GDP (R$)	GDP *per capita* (R$)
Barbalha	479.184 (2522°)	55,323 (545°)	115	0.683	499.981	8.934,61
Crato	1,009.202	135,604	129.41	0.713	1.478,136	11.578,96
Juazeiro	248	270,383	1,090.25	0.694	3.921,628	14.741,74
Crajubar	1,736.386 (863°)*	461,383 (50°)*	1,244.66	2.09	5.899,745 (150°)	35.255,31
Cariri	5,460.084	601,817 (35°)*	0.11	0.642	7.044,025	11.934

The position in the national ranking of municipalities with 5,570 municipalities is within parentheses, according to the Brazilian Institute of Geography and Statistics (IBGE - *Instituto Brasileiro de Geografia e Estatística*).

* Considering the hypothetical position in the ranking of the municipalities, in case the region was the sole municipality.

DD: demographic density; HDI: Human Development Index; GDP: Gross Domestic Product.

### Profile of claims

A total of 480 claims were obtained, excluding those related to compulsory hospitalizations. Table 2 data result from the first phase of the works to monitor outcomes.

**Table 2:** Surveys prior to the implementation of the Office

Type of claim	n	Not included in the SUS formulary (%)	Estimated cost R$ (%)	Cost of claims (%)
Medicines	163	26 (16)	436.057,56 (47.36)	47.3
Food	103	103 (100)	136.743,90 (14.85)	14.8
Surgeries	85	37 (43)	245.432,89 (26.66)	26.6
Tests	35	29 (83)	27.508,89 (2.99)	2.9
Other	32	20 (63)	74.996,99 (8.15)	8.1
Total	480	215 (45)	920.740,23	100
Checked outcomes		Occurrence		
Compliant prescriptions	201	-	276.809,87 (30.1)	41.9
Patients not found	115	74 data missing	380.044,98 (41.3)	24.0
		28 wrong addresses		
		13 never lived there		
Non-compliant prescriptions	96	-	150.331,57 (16.3)	20.0
Deaths	68	-	113.554,79 (12.3)	14.2
Total	480		920.740,23	100

SUS: Unified Health System.

The estimated cost corresponded to the value of the item not designated in the proceeding. Item “Medicines”, in table 2, implied values related to the periods of request mentioned in the proceedings; “surgeries and tests” corresponded to the values calculated based on three quotes obtained in the region at the same time. Non-compliant prescriptions referred to proceedings subject to discharge or changes in treatment, and the patients no longer used the medication mentioned in the proceeding. “Deaths” corresponded to deaths identified in the crossing of data and found in the active search. “Others” meant a set of items grouped together for analytical purposes.

### Claim profile

Amounts related to shelved proceedings were estimated based on market costs at the time of NAJS implementation, because only the prescription and the court decision were available. Surgery and test proceedings are ended after they are conducted. Most medications require a monthly provision of financial resources. Accordingly, the costs presented are an estimate for the total mapping period. Some resources used for death purposes were identified. It was assumed that after death no notification was made to the Health Department or the Public Defender's Office. However, chances are that resources have been received for an undefined period of time after the patient's death. Neither such occurrences nor their possible costs have been checked. All cases were referred to the Public Defender's Office, as well as the cases with non-compliant identification.

All proceedings were accompanied by their medical prescription and the respective disease classification (International Classification of Diseases, 10^th^ Review – ICD-10). However, no registrations were found for plaintiff's reevaluation requested by court order or the Department of Health. Ideally, outcomes, treatment and if the initial measures proposed had been adequate, should have been monitored. Nonetheless, the initial prescription of the proceedings was not subject to any kind of subsequent follow-up. Therefore, it is possible to say that less than half of the cases filed in court were compliant with prescriptions (42%), and almost half of the claims were not included on the official formularies (45%).

The following data show the profile of judicialization in the 12-month period before and after NAJS implementation.

The significant difference (p=0.01) between the volume of claims filed in court before and after NAJS implementation may be attributed to mediation to support a court decision.

Table 3 shows proceedings which were not prevented and the service provided by the NAJS at the Public Defender's Office. The volume of claims filed in court decreased. Compulsory hospitalizations were not considered.

**Table 3:** Sociodemographic profile and legal actions filed in the 12-month period before and after implementation of the service

Variables		Before	After	p value
		n (%)	n (%)	
Total of proceedings		114	69	
Age, years				
	Mean	52.9	51.1	0.45
	Standard deviation	25.3	22.1	
	Minimum	0.3	0.5	
	Maximum	93.0	89.0	
Sex				
	Female	42 (36.8)	32 (46.4)	0.175
	Male	72 (63.2)	36 (52.2)	
Occupation				
	Unemployed	18 (15.8)	21 (30.4)	0.064
	Employed	47 (41.2)	23 (33.3)	
	Retiree	49 (43.0)	25 (36.2)	
Location				
	Rural zone	41 (36.0)	30 (43.5)	0.312
	Urban zone	73 (64.0)	39 (56.5)	
Origin of the proceeding				
	Private lawyer	1 (0.9)	1 (1.4)	0.718
	Public Defender's Office	113 (99.1)	68 (98.6)	
Supplies				
	Medicines	75 (65.8)	40 (62.5)	0.003*
	Tests	11 (9.6)	4 (6.3)	
	Milk	13 (11.4)	4 (6.3)	
	Surgical procedure	7 (6.1)	1 (1.5)	
	Enteral diet	5 (4.4)	1 (1.5)	
	Diapers	1 (0.9)	1 (1.5)	
	Transport ticket/daily expenses	0 (0.0)	4 (6.3)	
	Two/more supplies required	2 (1.8)	9 (14.1)	
SUS incorporated				
	No	88 (77.2)	65 (94.2)	0.003*
	Yes	26 (22.8)	4 (5.8)	
Specialist requested				
	No	43 (37.7)	19 (27.5)	0.158
	Yes	71 (62.3)	50 (72.5)	

* Statistically significant difference, p=0.003.

SUS: Unified Health System.

An agreement was reached so that new claims were not admitted without prior technical consultation. In April 2017, it was possible to see that no case had been admitted.

No significant difference in the sociodemographic profile of plaintiffs was identified, but difference was found for supplies (p=0.003) and for the item requested being or not incorporated by SUS (p=0.003). The cost of items not incorporated by SUS was higher.

There was no significant difference when comparing groups in proceedings before and after the NAJS implementation and sociodemographic characteristics. The median values of the minimum monthly cost per proceeding showed that half of them had relatively low value. However, the maximum monthly amount of cost was reduced after NAJS implementation, showing a decrease of discrepant values.

Only the relation between the type of supplies and incorporation by SUS showed significant difference (p=0.003). The absence of difference in the remaining variables studied suggested homogeneity of the proceedings and requests, where the principle of equity was protected, *i.e*., attention and service were offered regardless of the region of applicant's residence, their occupation, sex, and others, and the need and evaluation of each case were considered.

The relation between type of input and age was significant (p=0.008) by analysis of variance (ANOVA). The *post-hoc* analysis in the Games-Howell test indicated that the application for more than one supply was recurrent among older applicants than in requests for food (p=0.018) and surgery (p=0.038). The remaining supplies were distributed similarly, regardless of the age.

Table 4 shows data related to the profile of global costs in the period.

**Table 4:** Comparison of total estimated costs

Requested Item	Cost before R$ (%)	Cost after R$ (%)	Before/after relation	Modification %
Pharmaceutical supply	305.653,26 (90.6)	206.050,64 (53.44)	0.67	-33
Tests	3.651,20 (1.08)	10.660,00 (2.76)	2.92	192
Hospital procedures	16.122,50 (4.78)	2.787,00 (0.72)	0.17	-83
Blocked resources	11.686,51 (3.46)	0		N/A
Other	0	13.560,00 (5.51)		N/A
Total	337.113,47	233.057,64	0.69	-31
Average cost per proceeding	2.957,14	3.377,65	1.14	14

The items of this table represent the universe of requests made within the period. Costs are presented in Brazilian *Reals*, calculated in April 2017. The percentage of costs refer to judicialization in the period. The percentage modification estimates the dimension of the modification in the scenario before and after NAJS implementation.

Judicialization cost of the items presented showed a global reduction of approximately 30%. The request for pharmaceutical supplies decreased in the amount requested (from 90% to 53%). Compulsory hospitalizations proceedings were ended upon hospitalization. Accordingly, in the survey before NAJS it was not possible to obtain such data. The increase in the average cost per proceeding by approximately 14% was also identified, suggesting that more costly items started to be requested.

## DISCUSSION

This study is the first of its kind to propose the active monitoring of claims filed in court, compliance, attainment and outcomes, according to plaintiff's knowledge. Said proposal had been formerly suggested,^(^^6^^)^ but this is the first work to have a direct follow-up, on a case by case basis, to check the outcomes.^(^^23^^)^ Increased efficiency is expected in the proceedings, both due to the monitoring of outcomes in health^(^^24^^)^ and also to prevent waste of resources due to negligence, improper use, embezzlement or other means.^(^^25^^)^

The data before the implementation show a scenario compatible with the problem. The data presented indicate that half of the proceedings examined had prescription problems, where accuracy was not taken into account. It was not feasible to assess the relevance or correction of the indications, since a medical team would be necessary to evaluate the prescription. Therefore, even data on “Prescription compliance” may be subject to discussion, as reported in other articles,^(^^26^^)^ and that would potentially disclose a more problematic situation, particularly if the relevance of scientific evidence is taken into account.^(^^11^^)^

In the first 6 months after NAJS implementation, the number of claims filed in court continued similar to the previous period. On the other hand, in the 6 months thereafter, there was a trend towards the decrease of claims, and also in the quantity of executed proceedings. The hypothesis is that as there is more information available for the interested individuals, they abstain from going to court, to their physicians and/or lawyers and are starting to seek administrative alternatives within SUS.

Regarding the comparison of judicialization costs in the periods before and after, it is possible to verify the decrease by approximately 30% in global costs. Together with the smaller volume of proceedings filed even after the request was granted, it is possible to suggest that the decrease in the financial impact of judicialization for the municipality was achieved. It should be emphasized that, despite the increase in the average cost per proceeding, it is possible to estimate that this fact is due to the increase in solutions found within SUS lists, and that is a positive aspect if compared to data from other authors.^(^^2^^)^ According to them, most lawsuits could have been prevented if therapeutic alternatives available on SUS formularies had been considered. This seems to be the case of part of NAJS success. On the other hand, most of the medicines requested are not included on SUS formularies, challenging the data of the same study.

A material aspect to be considered is the fact that statistical analyses have not shown significant differences among practically no subgroup of plaintiffs analyzed. The hypothesis here is that, despite all economic consequences of judicialization, no unequal distribution of resources has taken place for the populations sampled. The decrease in the costs of judicialization and the increase in the number of settlements which prevented judicial intervention did not take place to the detriment of any group or nature of claim. Said fact suggests that NAJS preserved the equitable treatment of claims of the social groups.

The service presented here was implemented with resources already existing in the municipality – personnel and physical implementations – for which reason it is possible to acknowledge the merit to represent a strategy applicable in municipalities of Brazil similar in its sociodemographic and economic characteristics. The management tools adopted in this study were efficient not only in financial terms, but in terms of improvements in the client service and strengthening of the health network, by means of the complete evaluation during home visits.

As study limitation it is possible to report cost estimate based on market researches. This type of estimates is more costly than those practiced by the government. Despite not having a standard financial reference, it was possible to verify the influence of the initiative in the global results. Other aspect refers to structural and registration limitations, which make the recovery of precise data difficult, since health care-related claims need to be solved on an urgent basis, and for this reason bidding processes are waived. For this reason, the cost estimate may vary considerably.

The specialized literature does not approach matters involving the final allocation of resources made available to the individual. It is the Judicial Branch's responsibility to issue a court order, but not to monitor its outcome. That is the duty of the Departments of Health. Most municipalities have no funds available for this type of monitoring. Therefore, the result of the resources allocated in legal actions are not followed up if there is no close collaboration of justice and health. This negatively affects the efficiency of the proceeding and is a challenge for the management of Government resources.

## CONCLUSION

This study proposed a feasible model to monitor outcomes of health-related claims filed in court for municipalities with similar characteristics to those of this study. The results obtained show a positive impact on the management of financial resources in health for making the health system and the user to come closer to each other, and for providing data for the judiciary system. The implementation of the Office for Assistance in Judicialization of Healthcare in the municipality of Barbalha (CE, Brazil), in partnership with the Public Defenders' Office allowed savings of the resources applied in lawsuits, and established ties among the municipality institutions. This finding means improvement of government resources, which is beneficial for the Unified Health System user.

## References

[1] 1. Paim LF, Batt CR, Saccani G, Guerreiro IC. Qual é o custo da prescrição pelo nome de marca na judicialização do acesso aos medicamentos? Cad Saude Colet. 2017;25(2):201-9.

[2] 2. Catanheide ID, Lisboa ES, Souza LE. Características da judicialização do acesso a medicamentos no Brasil: uma revisão sistemática. Physis. 2016; 26(4):1335-56.

[3] 3. Santos EC, Teixeira CR, Zanetti ML, Istilli PT, Pereira LH, Torquato MT. Health judicialization: access to treatment for users with diabetes mellitus. Texto Contexto Enferm. 2018;27(1):e0800016.

[4] 4. Campos Neto OH, Acurcio FA, Machado MA, Ferré F, Barbosa FL, Cherchiglia ML, et al. Doctors, lawyers and pharmaceutical industry on health lawsuits in Minas Gerais, Southeastern Brazil. Rev Saude Publica. 2012;46(5):784-90. 10.1590/s0034-89102012000500004 23128254

[5] 5. Pepe VL, Figueiredo Tde A, Simas L, Osorio-de-Castro CG, Ventura M. A judicialização da saúde e os novos desafios da gestão da assistência farmacêutica. Cien Saude Colet. 2010;15(5):2405-14. 10.1590/s1413-81232010000500015 20802873

[6] 6. Nunes CF, Ramos Júnior AN. Judicialização do direito à saúde na região Nordeste, Brasil: dimensões e desafios. Cad Saude Colet. 2016;24(2):192-9.

[7] 7. Wang DW. Right to health litigation in Brazil: the problem and the institutional responses. Hum Rights Law Rev. 2015;15(4):617-41.

[8] 8. Pepe VL, Ventura M, Sant'ana JM, Figueiredo TA, Souza Vdos R, Simas L, et al. Caracterização de demandas judiciais de fornecimento de medicamentos “essenciais” no Estado do Rio de Janeiro, Brasil. Cad Saude Publica. 2010;26(3):461-71. 10.1590/s0102-311x2010000300004 20464065

[9] 9. Nisihara RM, Possebom AC, Borges LM, Shwetz AC, Bettes FF. Judicial demand of medications through the Federal Justice of the State of Paraná. einstein (São Paulo). 2017;15(1):85-91. 10.1590/S1679-45082017GS3792 PMC5433313 28444095

[10] 10. Oliveira MR, Delduque MC, Sousa MF, Mendonça AV. Judicialização da saúde: para onde caminham as produções científicas? Saúde Debate. 2015; 39(105):525-35.

[11] 11. Dias ER, da Silva Junior GB. Evidence-Based Medicine in judicial decisions concerning right to healthcare. einstein (São Paulo). 2016;14(1):1-5. 10.1590/S1679-45082016AO3363 PMC4872909 27074226

[12] 12. Sant'ana JM, Pepe VL, Figueiredo TA, Osorio-de-Castro CG, Ventura M. Rational therapeutics: health-related elements in lawsuits demanding medicines. Rev Saude Publica. 2011;45(4):714-21. 10.1590/s0034-89102011005000042 21739079

[13] 13. Macedo EI, Lopes LC, Barberato-Filho S. A technical analysis of medicines request-related decision making in Brazilian courts. Rev Saude Publica. 2011;45(4):706-13. 10.1590/s0034-89102011005000044 21739077

[14] 14. Lopes LC, Barberato-Filho S, Costa AC, Osorio-de-Castro CG. Rational use of anticancer drugs and patient lawsuits in the state of São Paulo, Southeastern Brazil. Rev Saude Publica. 2010;44(4):620-8. 10.1590/s0034-89102010000400005 20676553

[15] 15. Victora CG, Barreto ML, do Carmo Leal M, Monteiro CA, Schmidt MI, Paim J, Bastos FI, Almeida C, Bahia L, Travassos C, Reichenheim M, Barros FC; Lancet Brazil Series Working Group. Health conditions and health-policy innovations in Brazil: the way forward. Lancet. 2011;377(9782):2042-53. 10.1016/S0140-6736(11)60055-X 21561659

[16] 16. São Paulo. Ministério Público do Estado de São Paulo. MPSP assina acordo com Secretaria da Saúde para elaborar mapa da judicialização [Internet]. São Paulo (SP); 2016 [citado 2018 Jun 31]. Disponível em: http://www.mpsp.mp.br/portal/page/portal/noticias/noticia?id_noticia=16110300&id_grupo=118

[17] 17. Brasil. Conselho Nacional de Justiça. Recomendação n. 31, de 30 de março de 2010. Recomenda aos Tribunais a adoção de medidas visando melhor subsidiar os magistrados e demais operadores do direito, para assegurar maior eficiência na solução das demandas judiciais envolvendo a assistência a saúde [Internet]. Brasília (DF): 2010 [citado 2018 Ago 23]. Disponível em: http://www.cnj.jus.br/atos-normativos?documento=877

[18] 18. Brasil. Conselho Nacional de Justiça. Recomendação n. 107, de 6 de abril de 2010. Institui o Fórum do Judiciário para monitoramento e resolução das demandas de assistência à saúde [Internet]. Brasília (DF); 2010 [citado 2018 Ago 23]. Disponível em: https://atos.cnj.jus.br/files/resolucao_107_06042010_11102012191858.pdf

[19] 19. Pereira JG, Pepe VL. Acesso a medicamentos por via judicial no Paraná: aplicação de um modelo metodológico para análise e monitoramento das demandas judiciais. Rev Dir Sanit. 2014;15(2):30-45.

[20] 20. Husereau D, Drummond M, Petrou S, Carswell C, Moher D, Greenberg D, et al. Consolidated health economic evaluation reporting standards (CHEERS) statement. BMJ. 2013;346:f1049. 10.1136/bmj.f1049 23529982

[21] 21. Lopes LM, Asensi FD, Silva Junior AG. The indirect judicialization of health: a case study on the experience of Cachoeiro de Itapemirim/ES. Rev Direito e Praxis. 2017;8(1):285-320.

[22] 22. Instituto Brasileiro de Geografia e Estatística (IBGE). Cidades e estados [Internet]. IBGE; 2018 [citado 2018 Ago 27]. Disponível em: https://cidades.ibge.gov.br/

[23] 23. Stamford A, Cavalcanti M. Legal decisions on access to medicines in Pernambuco, Northeastern Brazil. Rev Saude Publica. 2012;46(5):791-9. 10.1590/s0034-89102012000500005 23128255

[24] 24. Bittencourt GB. O “Estado da Arte” da produção acadêmica sobre o fenômeno da judicialização da saúde no Brasil. Cad Ibero-Amer Dir Sanit. 2016;5(1):102-21.

[25] 25. Diniz D, Machado TR, Penalva J. A judicialização da saúde no Distrito Federal, Brasil. Cien Saúde Colet. 2014;19(2):591-8. 10.1590/1413-81232014192.23072012 24863835

[26] 26. Camargo EB, Pereira AC, Gliardi JM, Pereira DR, Puga ME, Silva ET, et al. Judicialização da saúde: onde encontrar respostas e como buscar evidências para melhor instruir processos. Cad Ibero-Amer Dir Sanit. 2017;6(4):27-40.

