# Supplementary material for: Economic evaluation of an active search system to monitor the outcomes of health-related claims
Source: Einstein (Sao Paulo). 2019 Dec 20;18:eGS5129. doi: 10.31744/einstein_journal/2020GS5129 (PMC6924821; doi:10.31744/einstein_journal/2020GS5129)
Supplement: Translation

# Translation: Avaliação econômica de um sistema de busca ativa para monitoramento de desfechos em demandas de saúde judicializadas

**Authors:** Nayara Luiza Pereira Rodrigues, Victor Zaia, Joseval Martins Viana, Paulo Roberto do Nascimento, Erik Montagna

**Affiliations:** 1 Faculdade de Medicina do ABC, Centro Universitário Saúde ABC, Santo André, SP, Brasil.; 2 Superintendência de Controle de Endemias, Secretaria Estadual de Saúde, São Paulo, SP, Brasil.

**Keywords:** Judicialização da saúde, Equidade no acesso aos serviços de saúde, Avaliação de resultados (cuidados de saúde), Equidade na alocação de recursos

## Abstract

**Objetivo:**

Avaliação econômica de um programa de aconselhamento científico junto à defensoria pública para minimizar o impacto da judicialização da saúde no município, bem como da implementação de um programa de pesquisa ativa para monitorar os desfechos em saúde provenientes de demandas judiciais.

**Métodos:**

Estudo conduzido em duas etapas. A primeira foi documental, retrospectiva, e composta por dados coletados de processos judiciais de 2013 a 2018 da região de Barbalha, no estado do Ceará. A segunda etapa foi prospectiva e de intervenção, conduzida por meio da mediação entre o cidadão e a defensoria pública, com o objetivo de reduzir a ocorrência da judicialização e monitorar os resultados dos processos de saúde. O estudo adotou o protocolo para avaliações econômicas em saúde Roteiro para Relato de Estudos de Avaliação Econômica. Os dados obtidos foram agrupados e tratados para caracterização do cenário. Comparou-se o perfil dos processos no período de 12 meses antes e após a instalação do programa para delimitar ciclo fiscal completo.

**Resultados:**

O serviço de consultoria promoveu redução de 40% (p=0,01) nas ações judiciais. Além disso, observou-se redução de 31% nos custos judiciais (p=0,003) com a medicação sendo responsável por 33% desse valor. Observou-se redução no uso de insumos não constantes nas listas do Sistema Único de Saúde (27%; p=0,003), contudo, sem diferença estatística entre os grupos.

**Conclusão:**

Os dados desta pesquisa foram comparáveis aos já relatados em pesquisas brasileiras quanto ao perfil de demandas. A proposta mostrou-se viável como meio de mitigar os custos da judicialização por meio da mediação. Essa iniciativa pode servir como modelo para os municípios que possuem características similares às apresentadas em nosso estudo.

## INTRODUÇÃO

A judicialização da saúde caracteriza o acesso ao Sistema Único de Saúde (SUS) por meio de determinações judiciais, interferindo no cumprimento de políticas de saúde e ocorrendo de forma não programada e acima do orçamento previsto.^(^^1^^)^ Envolve questões éticas, como o papel do Estado na mediação dos interesses e direitos individuais e coletivo,^(^^2^^)^ em razão do desequilíbrio gerado no planejamento da saúde,^(^^3^^)^ prejudicando o atendimento dos beneficiários do sistema.^(^^4^^)^

Há expressivo volume de dados de pesquisas acadêmicas compreendendo requisições de medicamentos,^(^^5^^)^ características de processos,^(^^2^^)^ mapeamento das demandas judiciais em diversos cenários,^(^^6^^)^ dentre outros. Mesmo assim, os meios para obtenção de dados confiáveis são descentralizados, com acesso variável à informação e ausência de padronização,^(^^7^^)^ não obstante esforços empreendidos por grupos de pesquisa em segmentos específicos e com resultados expressivos.^(^^8^^)^

O principal item requerido judicialmente é o medicamento.^(^^9^^)^ Por outro lado, os desfechos raramente são abordados, e há reconhecida necessidade de maior aporte acadêmico-científico, para ampliar o suporte das decisões jurídicas.^(^^10^^)^

Tais decisões têm sido objeto de estudo, e admite-se haver elevada interferência judicial nas decisões,^(^^11^^)^ sobretudo por questões técnicas e científicas não observadas nestas decisões. Referem-se a receitas médicas que não atendem às boas práticas de prescrição,^(^^12^^)^ ainda que existam diretrizes legais para tanto; motivações dos demandantes na prescrição de medicamento por nome de marca,^(^^13^^)^ questionamentos quanto à eficácia de indicações terapêuticas^(^^14^^)^ e ausência de evidências científicas nas prescrições.^(^^11^^)^ Mesmo assim, a prescrição médica vigora como elemento soberano para suportar a sentença.^(^^11^^)^

Os conflitos de interesses também são debatidos, considerando que parcela expressiva das demandas parte do setor privado,^(^^15^^)^ estando concentradas em um número limitado de profissionais médicos ou advogados.^(^^6^^)^ Apesar de iniciativas governamentais para atenuar os custos de fraudes, corrupção e desvios de recursos, valores expressivos são desperdiçados.^(^^16^^)^ Entretanto, casos desta natureza devem ser tratados no âmbito criminal.

Este complexo cenário motivou o Conselho Nacional de Justiça (CNJ) a incentivar que Tribunais Federais e Estaduais buscassem apoio técnico para a formação de juízo nas ações envolvendo questões relativas à saúde, e à formulação de resoluções para monitoramento e solução de demandas de assistência à saúde.^(^^17^^,^^18^^)^

Assim, o Poder Público tem meios para que a judicialização não seja a primeira opção do usuário do SUS. Dentre eles, estão a intervenção que evite o início do processo judicial por meio de assessoria científica, as conciliações antes do ajuizamento das ações evitando a judicialização e o monitoramento de desfechos em busca de eficiência no processo em toda sua extensão.^(^^19^^)^

Entretanto, recursos em tecnologia da informação não estão disponíveis para a maioria dos municípios brasileiros. Monitorar o destino dos recursos, seus usos e desfechos não é contemplado atualmente. Assim, apesar do controle e da rastreabilidade destes processos no fornecimento dos recursos, não há monitoramento dos resultados do investimento. A atomização destes recursos alcança indivíduos pela capilaridade do sistema e impõe um desafio na gestão, ao criar a demanda de monitoramento pontual e personalizado do sistema.

## OBJETIVO

Realizar a avaliação econômica do Núcleo de Assistência da Judicialização na Saúde, promovido pela Secretaria Municipal de Saúde na Defensoria Pública, que visa minimizar os impactos da judicialização da saúde em um município brasileiro. A partir desses dados, propõe-se o Núcleo de Assistência da Judicialização na Saúde como modelo de atuação para municípios com características sociodemográficas similares.

## MÉTODOS

### Desenho experimental

Estudo documental, retrospectivo, exploratório, descritivo, quantitativo, realizado a partir da coleta de dados das ações judiciais deferidas para acesso ao sistema de saúde pleiteados na comarca de Barbalha (CE), Brasil. Adotou-se o protocolo *Consolidated Health Economic Evaluation Reporting Standards* (CHEERS), para padronização de apresentação de resultados de avaliações econômicas em saúde.^(^^20^^)^

### Procedimentos

Descrição da implementação do Núcleo de Atendimento de Judicialização da Saúde (NAJS) e suas atribuições, comparando os gastos com a judicialização antes (Cenário A) e depois (Cenário B) de sua implantação. Foi realizado o mapeamento das demandas judiciais, suas repercussões para os atendimentos e o levantamento dos perfis dos requerentes e das requisições. Também foram estimados os custos dos casos judicializados. As comparações compreenderam 12 meses antes e depois de sua instalação, visando circunscrever a um período de tempo que permitisse a realização de comparações quantitativas. Esperou-se contemplar um ciclo completo de variações sazonais e um período comparável ao ano fiscal. Por fim, foram descritas as estratégias de gestão aplicadas para evitar a judicialização. O universo do estudo foi constituído por todos os processos impetrados contra a Secretaria de Saúde no período de janeiro de 2013 até abril de 2018. A extração de dados foi realizada manualmente.

### Mapeamento de cenário

O município está localizado na Região Metropolitana do Cariri, parte da conurbação dos municípios de Crato e Juazeiro do Norte.

O NAJS foi criado em abril de 2017, com uma equipe composta por uma enfermeira, um farmacêutico e uma assistente social. Dentre suas atribuições, duas implementadas na primeira fase de funcionamento foram o objeto deste trabalho:

– Monitorar os desfechos das demandas de saúde judicializadas no município; verificar seu cumprimento do ponto de vista jurídico; detectar e evitar fraudes e desperdícios, e obter dados relativos à necessidade ou não da manutenção do benefício.
– Mediar a relação entre usuário demandante e sistema judiciário, propondo soluções por vias administrativas no SUS que evitassem a judicialização.

O atendimento de demandas ocorre com a presença dos três profissionais do NAJS, o Defensor Público, o requerente e seus representantes legais. É realizado um cadastro com dados pessoais, sociodemográficos, motivo e tipo de demanda, exames e intervenções já realizados, indicação médica para a solicitação – todos acompanhados de documentos comprobatórios –, quando ocorreu a última visita do Agente Comunitário de Saúde e a última avaliação de saúde realizada por especialista. Em um prazo de 7 dias é dada resposta ao usuário sobre o procedimento a ser seguido, que evite a judicialização.

### Processos analisados

Foram analisados todos os processos que envolveram demandas judiciais em saúde no município entre janeiro 2013 até abril de 2018 que não estivessem sob segredo. A identificação e a classificação dos processos foram realizadas por meio do Sistema de Automação do Poder Judiciário do Estado do Ceará (e-Jud), mediante consultas efetivadas por servidores autorizados.^(^^21^^)^ Os dados de acompanhamento de desfechos estão relacionados a processos judicializados e, desse modo, estão publicamente disponíveis. Foram preservadas as identidades dos requerentes, advogados, médicos e quaisquer envolvidos mencionados nos processos.

### Estimativas de custos

As estimativas de custos foram baseadas em trabalhos anteriormente publicados.^(^^1^^)^ O valor médio de cada unidade farmacológica, insumo e procedimento foi estimado por pesquisa de mercado e pela planilha de preços máximos ao governo. Para o cálculo do valor unitário dos medicamentos, foi utilizada uma apresentação de cada laboratório produtor com o maior número disponível de formas farmacêuticas. Visou-se à estimativa do valor unitário médio de cada unidade farmacológica para cada um dos tratamentos para o período do estudo, bem como para os insumos e procedimentos.

### Busca ativa

Após a avaliação e a coleta de dados de todos os processos, o NAJS buscou os desfechos dos processos em aberto. Casos que pudessem ser dirimidos por meio de cruzamentos de dados públicos oficiais foram realizados em escritório (por exemplo: óbito dos requerentes). Os demais casos foram visitados pelo NAJS. Todos os casos em que foram encontradas não conformidades foram oficiados e encaminhados à Defensoria Pública para providências legais.

Usuários do SUS atendidos por meio judicial foram visitados. Foram verificados endereço, dados do item demandado e da manutenção ou alteração da condição de saúde do requerente. Em caso de alteração da condição de saúde que mudasse o *status* do que fora obtido judicialmente, era solicitado ao requerente seu comparecimento na Secretaria de Saúde. Após a verificação de todos os processos analisados, o NAJS passou a acompanhar somente os casos em andamento e que estivessem em conformidade com as prescrições médicas e legais.

### Análise de dados

Para as análises estatísticas, foi utilizado o programa (SPSS; IBM, 2010), versão 20.0 para Macintosh. Os dados foram agrupados segundo as variáveis disponíveis nos processos. Foram realizadas análises descritivas a partir da apuração de frequências, com sumarização dos resultados. Foram utilizadas estatísticas descritivas (média e desvio padrão), correlacionais (rhô de Spearman) e U de Mann-Whitney, quando dois grupos foram comparados, e o teste de Kruskall-Wallis, para três ou mais grupos. Para o tipo de regressão adequada aos dados, foi verificada a distribuição da normalidade das variáveis estudadas pelo teste de Kolmogorov-Smirnov.

### Aspectos éticos

O trabalho foi realizado segundo as normas do Conselho Nacional de Saúde. Por tratar-se de estudo documental envolvendo processos judiciais, foi solicitada dispensa da aplicação do Termo de Consentimento Livre e Esclarecido (TCLE). O estudo foi aprovado pelo Comitê de Ética em Pesquisa, parecer 2.924.500, CAAE: 98718918.8.0000.0082, e autorizado pela Secretaria Municipal de Saúde e pela Defensoria Pública de Barbalha (CE, Brasil).

## RESULTADOS

### Caracterização de cenário *(setting)*

A cidade de Barbalha ocupava a sétima colocação no estado em Índice de Desenvolvimento Humano (IDH) geral, a nona em IDH-Educação e a quarta em IDH-Longevidade.^(^^22^^)^ As características sociodemográficas regionais estão apresentadas na tabela 1.

**Tabela 1:** Caracterização sociodemográfica da microrregião do Cariri

Município	Área (km^2^)	Habitantes	DD (hab/km)	IDH	PIB (R$)	PIB *per capita* (R$)
Barbalha	479,184 (2522°)	55.323 (545°)	115	0,683	499.981	8.934,61
Crato	1.009,202	135.604	129,41	0,713	1.478,136	11.578,96
Juazeiro	248	270.383	1.090,25	0,694	3.921,628	14.741,74
Crajubar	1.736,386 (863°)*	461.383 (50°)*	1.244,66	2,09	5.899,745 (150°)	35.255,31
Cariri	5.460,084	601.817 (35°)*	0,11	0,642	7.044,025	11.934

Entre parênteses está a posição ocupada no *ranking* nacional de municípios, que conta com 5.570 municípios segundo o Instituto Brasileiro de Geografia e Estatística.

* Considerando a posição hipotética ocupada no *ranking* de municípios, caso a região fosse um único município.

DD: densidade demográfica; IDH: Índice de Desenvolvimento Humano; PIB: Produto Interno Bruto.

### Perfil das demandas

Foram obtidos 480 processos, excluídos os referentes às internações compulsórias. Os dados da tabela 2 são resultado da primeira fase de trabalhos de monitoramento de desfechos.

**Tabela 2:** Levantamentos anteriores à instalação do Núcleo

Tipo de demanda	n	Fora da lista do SUS (%)	Custo estimado R$ (%)	Custo processos (%)
Medicamentos	163	26 (16)	436.057,56 (47,36)	47,3
Alimentação	103	103 (100)	136.743,90 (14,85)	14,8
Cirurgias	85	37 (43)	245.432,89 (26,66)	26,6
Exames	35	29 (83)	27.508,89 (2,99)	2,9
Outros	32	20 (63)	74.996,99 (8,15)	8,1
Total	480	215 (45)	920.740,23	100
Desfechos verificados		Ocorrência		
Concordantes com prescrição	201	-	276.809,87 (30,1)	41,9
Pacientes não localizados	115	74 dados faltantes	380.044,98 (41,3)	24,0
		28 endereços errados		
		13 nunca moraram no local		
Discrepantes da prescrição	96	-	150.331,57 (16,3)	20,0
Óbitos	68	-	113.554,79 (12,3)	14,2
Total	480		920.740,23	100

SUS: Sistema Único de Saúde.

O custo estimado correspondeu ao valor do item não designado no processo. O item “medicamentos”, na tabela 2, implicou em valores apresentados referentes aos períodos de solicitação indicados nos processos; “cirurgias e exames” equivaleram aos valores calculados com base em três cotações de valores na região na mesma época. Discrepantes da prescrição se relacionou aos processos em que houve alta ou mudança do tratamento, e os pacientes não faziam mais uso da medicação citada no processo.

Os óbitos correspondem a óbitos identificados no cruzamento de dados e os apurados na busca ativa. Foi designado como “outros” o conjunto variado de itens para os quais optou-se pelo agrupamento para fins analíticos.

### Perfil das demandas

A estimativa de valores dos processos arquivados foi feita com base em custos de mercado na época da instalação do NAJS, pois existem apenas a prescrição e a ordem de cumprimento judicial. Cirurgias e exames têm o processo encerrado após sua realização. As medicações, em sua maioria, necessitam de aporte mensal de recursos. Desta forma, os custos apresentados são uma estimativa para o período total do mapeamento. Houve identificação de recursos destinados em alguns casos de óbito. Considerou-se que, após o óbito, não houve notificação da Secretaria de Saúde e nem da Defensoria Pública. Entretanto, é possível que tenha ocorrido recebimento de recursos por tempo não determinado após o falecimento do paciente. Não foram verificadas tais ocorrências nem estimados seus possíveis custos. Todos os casos foram encaminhados à Defensoria Pública, assim como os casos em que ocorreram discrepâncias de identificação.

Em todos os processos foram localizadas a prescrição médica e a respectiva classificação de doenças (Código Internacional de Doenças, 10^a^ Revisão – CID-10). Entretanto, não foram encontrados registros para reavaliação do demandante, nem por solicitação da justiça, nem por parte da Secretaria de Saúde. Idealmente, deveria existir acompanhamento do desfecho, do tratamento ou se as medidas inicialmente propostas mantinham-se adequadas. Verificou-se, entretanto, que a prescrição inicial dos processos não previa qualquer acompanhamento posterior. Assim, destaca-se que menos da metade dos casos judicializados estavam em concordância com a prescrição (42%), e quase metade das demandas não constavam nas listas oficiais (45%).

Os dados a seguir mostram o perfil de judicialização no período de 12 meses pré e pós-instalação do NAJS.

A diferença significativa (p=0,01) entre o volume de demandas judicializadas antes e depois do NAJS pode ser atribuída à mediação para racionalizar a decisão judicial.

Na tabela 3, são apresentados processos que não foram evitados e os atendimentos realizados pelo NAJS na Defensoria Pública. Houve diminuição no volume de demandas judicializadas. Foram desconsideradas as internações compulsórias.

**Tabela 3:** Perfil sociodemográfico e de demandas judiciais no período de 12 meses pré e pós instalação do serviço

Variáveis		Pré	Pós	Valor de p
		n (%)	n (%)	
Total de processos		114	69	
Idade, anos				
	Média	52,9	51,1	0,45
	Desvio padrão	25,3	22,1	
	Mínimo	0,3	0,5	
	Máximo	93,0	89,0	
Sexo				
	Feminino	42 (36,8)	32 (46,4)	0,175
	Masculino	72 (63,2)	36 (52,2)	
Ocupação				
	Desempregado	18 (15,8)	21 (30,4	0,064
	Empregado	47 (41,2)	23 (33,3)	
	Aposentado	49 (43,0)	25 (36,2)	
Localização				
	Zona rural	41 (36,0)	30 (43,5)	0,312
	Zona urbana	73 (64,0)	39 (56,5)	
Origem processo				
	Advogado particular	1 (0,9)	1 (1,4)	0,718
	Defensoria pública	113 (99,1)	68 (98,6)	
Insumos				
	Medicamentos	75 (65,8)	40 (62,5)	0,003*
	Exames	11 (9,6)	4 (6,3)	
	Leite	13 (11,4)	4 (6,3)	
	Procedimento cirúrgico	7 (6,1)	1 (1,5)	
	Dieta enteral	5 (4,4)	1 (1,5)	
	Fraldas	1 (0,9)	1 (1,5)	
	Passagem/diária	0 (0,0)	4 (6,3)	
	Dois/mais insumos requeridos	2 (1,8)	9 (14,1)	
Incorporado SUS				
	Não	88 (77,2)	65 (94,2)	0,003*
	Sim	26 (22,8)	4 (5,8)	
Solicitado especialista				
	Não	43 (37,7)	19 (27,5)	0,158
	Sim	71 (62,3)	50 (72,5)	

* Diferença estatisticamente significante, p=0,003.

SUS: Sistema Único de Saúde.

Houve um acordo para que novas demandas não fossem acatadas sem consultoria técnica. Naquele momento, em abril de 2017, foi possível verificar que nenhum caso foi atendido.

Não houve diferença significativa no perfil sociodemográfico dos demandantes. Entretanto, houve diferença para insumos (p=0,003) e quanto ao item demandado estar ou não incorporado ao SUS (p=0,003). Quando o item não esteve incorporado ao SUS, seu custo foi mais elevado.

Não houve diferença significativa nas comparações entre os grupos nos processos anteriores e posteriores à implantação do NAJS e características sociodemográficas. A mediana dos valores de custo mensal mínimo por processo mostrou que metade deles possuía valor relativamente baixo. Contudo, houve redução do valor máximo mensal de custo após a implantação do NAJS, apontando para diminuição nos valores discrepantes.

Somente a relação entre tipo de insumo e incorporação ao SUS mostrou diferença significativa (p=0,003). A ausência de diferença nas demais variáveis estudadas sugeriu homogeneidade dos processos e requisições, preservando o princípio de equidade. Ou seja, atenção e atendimento ocorreram independentemente da região de moradia, de quem realizava o pedido, da ocupação, sexo e outros, vinculando-se à necessidade e à avaliação de cada caso.

A relação entre tipo de insumo e idade foi significativa (p=0,008) pela análise de variância (ANOVA). A análise *post-hoc* no teste de Games-Howell indicou que o pedido de mais de um insumo foi mais recorrente entre requerentes mais velhos do que no pedido de alimentação (p=0,018) e cirurgia (p=0,038). Os demais insumos se distribuíram de forma semelhante independentemente da idade.

Na tabela 4 são apresentados os dados referentes ao perfil de custos globais no período.

**Tabela 4:** Comparação de custos totais estimados

Requerido	Custo pré R$ (%)	Custo pós R$ (%)	Relação pós/pré	Alteração %
Insumo farmacêutico	305.653,26 (90,6)	206.050,64 (53,44)	0,67	-33
Exames	3.651,20 (1,08)	10.660,00 (2,76)	2,92	192
Procedimentos hospitalares	16.122,50 (4,78)	2.787,00 (0,72)	0,17	-83
Recursos bloqueados	11.686,51 (3,46)	0		N/A
Outros	0	13.560,00 (5,51)		N/A
Total	337.113,47	233.057,64	0,69	-31
Custo médio por processo	2.957,14	3.377,65	1,14	14

Os itens da tabela representam o universo de solicitações realizadas no período. Os custos estão apresentados em Reais, apurados em abril de 2017. Os percentuais dos custos referem-se à quanto representam da judicialização no período. A alteração percentual estima a dimensão da alteração no quadro pré e pós-NAJS.

Houve redução global em torno de 30% nos custos com judicialização dos itens apresentados. O requerimento de insumos farmacêuticos apresentou diminuição no montante requerido (de 90% para 53%). Internações compulsórias tiveram seus processos extintos quando foram executadas. Assim, no levantamento pré-NAJS não foi possível obter tais dados. Outro dado verificado foi o aumento do custo médio por processo em torno de 14%, sugerindo que itens mais caros passaram a ser requeridos.

## DISCUSSÃO

O presente trabalho é o primeiro a propor uma iniciativa de acompanhamento ativo das demandas instaladas pela judicialização, seu cumprimento, consecução e desfechos, segundo o conhecimento dos autores. Tal proposta foi sugerida anteriormente,^(^^6^^)^ mas este é o primeiro a ter um acompanhamento direto, processo a processo, para verificação dos desfechos.^(^^23^^)^ A estimativa é de aumento da eficiência do processo, seja por monitorar os desfechos em saúde,^(^^24^^)^ como para evitar desperdício de recursos, por negligência, mau uso, descaminho ou outros meios.^(^^25^^)^

Os dados presentes no mapeamento pré-instalação mostram quadro condizente com o problema. Os dados apresentados indicam que metade dos processos verificados apresentavam problemas de prescrição, sem que se levasse em conta sua precisão. Não foi exequível a avaliação da pertinência ou correção das indicações, pois requereria uma junta médica para julgar a prescrição. Portanto, mesmo os dados sobre a “Concordância com a prescrição” podem ser discutíveis, como já verificado em outros trabalhos,^(^^26^^)^ o que potencialmente revelaria uma situação mais problemática, sobretudo se for considerada a relevância da evidência científica.^(^^11^^)^

Nos primeiros 6 meses após a instalação do NAJS, o número de demandas judicializadas se manteve similar ao período anterior. Já nos 6 meses seguintes, houve tendência de diminuição das demandas, bem como da quantidade de processos executados. A hipótese é de que há mais informação para o demandante, que deixa de procurar as instâncias judiciárias, ou até mesmo seus médicos e/ou advogados, e passam a procurar meios dentro do próprio SUS para as alternativas administrativas.

Com relação à comparação dos custos da judicialização nos períodos pré e pós, é possível verificar diminuição em torno de 30% dos custos globais. Associado ao menor volume de processos que ocorrem mesmo após o atendimento do requerente, é possível sugerir que houve êxito na diminuição do impacto financeiro da judicialização para o município. Cabe ressaltar que, apesar do aumento do custo médio por processo, é possível estimar que este fato seja devido ao aumento de soluções dentro das listas do SUS, o que constitui aspecto positivo e comparável a dados de outros autores.^(^^2^^)^ egundo estes, a maioria das ações teria sido evitada caso fossem observadas as alternativas terapêuticas presentes nas listas do SUS. Este parece ser o caso de parte do êxito do NAJS. Por outro lado, a maior parte dos medicamentos requeridos não está incluída nas listas do SUS, contrariando dados do mesmo estudo.

Um aspecto relevante a ser ressaltado é o fato de as análises estatísticas não terem mostrado diferenças significativas entre praticamente nenhum subgrupo de demandantes analisados. A hipótese aqui levantada é que, apesar de todas as consequências econômicas da judicialização, não houve distribuição desigual dos recursos para as populações amostradas. A diminuição dos custos da judicialização e o aumento da quantidade de acordos que evitaram a intervenção judicial não se impuseram em detrimento de nenhum grupo ou natureza de demanda. Tal fato sugere que a atuação do NAJS preservou o tratamento equânime das demandas dos grupos sociais.

O serviço aqui apresentado se efetivou com recursos já instalados no município – pessoal e instalações físicas – diante do que pode-se conferir-lhe o mérito de vir a representar estratégia aplicável em municípios do Brasil similares por suas características sociodemográficas e econômicas. As ferramentas de gestão adotas nesse estudo foram eficazes não só em termos financeiros, mas em termos de melhorias de atendimento ao paciente e fortalecimento da rede de saúde, por meio da avaliação integral durante as visitas domiciliares.

Como limitações do estudo, pode-se mencionar a estimativa de custos, baseados em pesquisas de mercado. Estimativas desta natureza são mais caras do que as praticadas pelos governos. Mesmo sem um comparador financeiro padrão, foi possível verificar a influência da iniciativa nos resultados globais. Outro ponto são as limitações estruturais e de registro, que dificultam a recuperação de dados precisos, uma vez que as demandas em saúde são atendidas em caráter de urgência, o que dispensa o processo de licitação. Assim, a estimativa de custos pode ter variabilidade considerável.

A literatura especializada não aborda questões envolvendo o destino final dos recursos fornecidos ao indivíduo. Cabe ao Poder Judiciário a expedição da ordem judicial, mas não lhe cabe o monitoramento do desfecho, que é incumbência das Secretarias de Saúde. Parcela significativa de municípios não contam com recursos para este monitoramento. Assim, o resultado dos recursos empenhados nos processos judicializados não tem acompanhamento se não houver estreita colaboração entre a justiça e a saúde. Isto compromete a eficiência do processo e constitui um desafio de gestão dos recursos empenhados pelo Estado.

## CONCLUSÃO

O presente estudo propôs um modelo exequível para monitoramento de desfechos em demandas de saúde judicializadas para municípios que tenham características similares ao estudado. Os resultados obtidos mostram impacto positivo na gestão de recursos financeiros na saúde, por aproximar o sistema de saúde e seu usuário, e por fornecer dados para o sistema judiciário. A instalação do Núcleo de Apoio à Judicialização na Saúde no município de Barbalha (CE, Brasil), em parceria com a Defensoria Pública, propiciou economia de recursos investidos em processos judiciais e estreitou as relações entre as instituições do município. Tal achado constitui aprimoramento na gestão de recursos públicos, o que é benéfico para o próprio usuário do SUS.
